# Genetic and Non-Genetic Factor-Adjusted Association between Coffee Drinking and High-Density Lipoprotein Cholesterol in Taiwanese Adults: Stratification by Sex

**DOI:** 10.3390/nu11051102

**Published:** 2019-05-17

**Authors:** Tsui-Wen Hsu, Disline Manli Tantoh, Kuan-Jung Lee, Oswald Nfor Ndi, Long-Yau Lin, Ming-Chih Chou, Yung-Po Liaw

**Affiliations:** 1 Institute of Medicine, Chung Shan Medical University, Taichung 40201, Taiwan; irb@cgh.org.tw; 2 Superintendent Office, Cathay General Hospital, Taipei 106, Taiwan; 3 Department of Public Health, Institute of Public Health, Chung Shan Medical University, Taichung 40201, Taiwan; tantohdisline@yahoo.com (D.M.T.); jasminemachi@gmail.com (K.-J.L.); nforoswald2@yahoo.com (O.N.N.); 4 School of Medicine, Chung Shan Medical University, Taichung 40201, Taiwan; xillin681113@gmail.com; 5 Department of Family and Community Medicine, Chung Shan Medical University Hospital, Taichung 40201, Taiwan

**Keywords:** HDL-C, coffee drinking, sex, Taiwan Biobank, rs1800588, rs180077

## Abstract

Low high-density lipoprotein cholesterol (HDL-C) is a major risk factor for cardiovascular diseases (CVDs), the leading cause of global mortality. We aimed to determine the effect of coffee drinking and sex and their interaction, as well as rs1800588 and rs1800775 polymorphisms on HDL-C levels in Taiwanese adults. Data of 4262 men and 4813 women, aged 30–70 years, were retrieved from Taiwan Biobank. The interaction between sex and coffee drinking on HDL-C was significant (*p* = 0.0452). Coffee consumption was significantly associated with higher HDL-C levels in only women (β = 0.81679; *p* = 0.0246). However, rs1800588 and rs1800775 variants were significantly associated with HDL-C in both sexes. In women, β-values were 0.99080; *p* = 0.0059 and 3.16277; *p* < 0.0001 for rs1800588 CT and TT genotypes, respectively and −1.80954; *p* < 0.0001 and −2.81512; *p* < 0.0001 for rs1800775 AC and CC genotypes, respectively. In men, β-values were 1.32430; *p* < 0.0001 and 3.24976; *p* < 0.0001 for rs1800775 CT and TT genotypes, respectively and −1.96232; *p* < 0.0001 and −2.71536, *p* < 0.0001 for the AC and CC genotypes, respectively. In conclusion, coffee drinking was significantly associated with higher high-density lipoprotein (HDL) levels in women but not men after adjusting for confounders including rs1800588 (LIPC) and rs1800775 (CETP) variants.

## 1. Introduction

Cardiovascular diseases (CVDs) are among the leading causes of global mortality [1,2,3]. High-density lipoprotein cholesterol (HDL-C) levels play several clinically relevant roles in cardiovascular health. Lower levels of HDL-C are associated with cardiovascular disease morbidity and mortality [1,4,5]. However, higher levels protect against atherosclerosis and other CVDs [6,7]. It has been projected that a unit increase in HDL-C is associated with 1.9–2.3% and 3.2% reduction in the risk of CVDs in men and women, respectively [8]. The anti-atherosclerotic property of HDL-C is attributed to its ability to enhance the reverse cholesterol transport (RCT) pathway [6,7,9]. RCT is the removal of excess cholesterol from macrophages to the liver for excretion or redistribution to other tissues [6,10]. Other clinical benefits of HDL-C include the protection against apoptosis, inflammation, infections, thrombosis and oxidation [9,10]. 

Both genetic and non-genetic factors account for individual variability in high-density lipoprotein (HDL) levels.

About 40–60% of this variability is of genetic origin [11]. In general, HDL-C levels in women are higher than in men [12,13,14]. The minimal threshold levels are <40 mg/dL in men and <50 mg/dL in women [15,16]. The hepatic lipase (LIPC) and cholesterol ester transfer protein (CETP) genes are well established genetic factors that influence HDL-C levels and metabolism, as well as atherosclerosis [17]. The variants rs1800588 (LIPC) and rs100775 (CETP) have been respectively associated with higher and lower HDL-C levels [17,18,19,20]. The cardio-protective property of the LIPC gene is due to its potential to stimulate reverse cholesterol transport and clearance of intermediate-density lipoprotein (IDL) from circulation [21,22]. However, deficiency in LIPC favors the accumulation and circulation of cholesterol-rich atherogenic particles [17]. CETP promotes the transfer of cholesteryl ester from HDL-C to very low-density lipoprotein (VLDL), which is rich in triglycerides [22,23,24]. Its deficiency is thought to be a major reason for high levels of HDL-C in Asians [23]. 

Non-genetic factors including, age, exercise, diet, smoking, BMI, alcohol and coffee drinking play pivotal roles in modifying HDL-C levels and managing cardiovascular disease risk [1,14,25]. Coffee, a modifiable lifestyle factor is the second leading non-alcoholic beverage that is consumed worldwide [1]. So far, its association with HDL-C and CVDs is inconsistent. Hence, its role in cardiovascular health is still controversial [1,26]. For instance, coffee has been positively associated with HDL-C [27,28], implying that it might protect against CVDs. The antiatherogenic properties are ascribed to its antioxidant-rich phenolic compounds [7,27], which enhance HDL-C-mediated cholesterol efflux from macrophages [7]. However, some studies reported that coffee drinking and HDL-C are negatively related [26] while others reported no significant associations [29,30]. The association between coffee consumption and HDL-C appears to vary by sex. For instance, significant associations between coffee drinking and HDL-C were observed in women but not men [1]. Furthermore, drinking coffee was inversely associated with coronary calcification especially in women [1].

In most previously conducted studies, adjustments were not made for potential confounders like single nucleotide polymorphisms (SNPs), smoking, tea and alcohol drinking and diet, among others. Since the association between coffee drinking and HDL-C is inconsistent, there is a need for more investigations. Hence, this study was undertaken to explore the effect of coffee drinking and sex and their interaction on HDL-C in Taiwanese adults with rs1800588 (LIPC) and rs1800775 (CEPT) single nucleotide polymorphisms.

## 2. Methods

Genetic and non-genetic data of 9075 adult Taiwanese comprising 4262 men and 4813 women, aged 30–70 years, were obtained from the Taiwan Biobank, a national health resource, which possesses genetic information of over 200,000 ethnic Taiwanese inhabitants. All the biobank enrolees usually sign informed consent forms prior to the collection of data. Data on coffee, tea and alcohol consumption, age, sex, diet, exercise, smoking, second-hand smoke and disease history (diabetes and hypertension) were collected through questionnaires. The body mass index (BMI) and waist-hip ratio (WHR) were determined through physical examinations while high-density lipoprotein cholesterol (HDL-C), low-density lipoprotein cholesterol (LDL-C), triglyceride (TG) and rs1800588 (LIPC) and rs180077 (CEPT) single nucleotide polymorphisms (SNPs) were determined through biochemical examinations. 

Participants were classified as coffee drinkers if they drank coffee three or more times in a week. The mean menopausal age was 49.10 years. Hence, age was stratified into two as <49.10 and ≥49.10 years. Tea drinkers were those who drank tea at least once a day. Vegetarians included those who maintained a vegetarian diet at least six months before the data collection date. Those who did exercise for more than 150 min per week were categorized under the regular exercise group. BMI categories included underweight (BMI < 18.5 kg/m^2^), normal (18.5 ≤ BMI < 24 kg/m^2^), overweight (24 ≤ BMI < 27 kg/m^2^) and obesity (BMI ≥ 27 kg/m^2^). Non-smokers included those who never smoked or did not continuously smoke for six months or more. Former smokers were those who continuously smoked for a minimum of six months but were not smoking at the time that data were collected, while current smokers included those who ceaselessly smoked for six months or more and were still smoking. Exposure to second-hand smoke was defined as being exposed for at least 5 min per hour. Alcohol consumption categories comprised non-drinkers; those who did not drink alcohol or drank <150 cc per week for six months, former drinkers; those who quit alcohol drinking for more than six months and current drinkers; those whose weekly alcohol drinking for six consecutive months was at least 150 cc [31]. Ethical approval for this study was obtained from the institutional review board (IRB) of Chung Shan Medical University (CS2-16114).

### Statistical Analyses

HDL-levels in men and women were compared using the t-test and presented as means ± standard errors (SEs). Categorical variables, e.g., coffee drinking in male and female participants were compared using the chi-square test and presented as percentages. The association between coffee drinking and HDL-C was determined using multivariate linear regression models. Adjustments were made for rs1800588 and rs180077 genotypes, age, vegetarian diet, exercise, BMI, smoking, second-hand smoke, alcohol and tea drinking, WHR, LDL-C, TG and history of diabetes and hypertension. All the above statistical analyses were performed using the SAS 9.4 software (SAS Institute, Cary, NC, USA).

## 3. Results

The basic characteristics of the male and female participants are shown in Table 1. The mean (±SE) HDL-C levels in men (48.585 ± 0.191 mg/dL) and women (58.467 ± 0.171 mg/dL) were significantly different (*p* < 0.001). Among men, 1339 (31.42%) were coffee drinkers while 2923 (68.58%) were non-drinkers. Among women, 1661 (34.51%) were coffee drinkers and 3152 (65.49%) were non-drinkers. The association between coffee drinking and HDL-C in all participants is shown in Table 2. The male sex was significantly associated with lower levels of HDL-C compared to the female sex (β = −6.33560; *p* < 0.001) while coffee drinking was not significantly associated with HDL-C. However, there was a significant interaction between sex and coffee drinking (*p* = 0.0399). Variants of LIPC (rs1800588) and CETP (rs1800775) were significantly associated with higher and lower levels of HDL-C, respectively (Table 2). For rs1800588, the beta coefficients (β) were 1.14306 (*p* < 0.0001) and 3.24816 (*p* < 0.0001) for the CT and TT genotypes, respectively. For rs1800775, β-values were −1.79140 (*p* < 0.0001) and −2.71185 (*p* < 0.0001) for the AC and CC genotypes, respectively (Table 2). After stratification by sex, there was no significant association between coffee consumption and HDL-C in men (Table 3). However, the rs1800588 genotypes remained significantly associated with higher HDL-C levels in men (β = 1.32430; *p* < 0.0001 for CT and β = 3.24976; *p* < 0.0001 for TT) while the rs1800775 genotypes remained significantly associated with lower levels of HDL-C (β = −1.80954; *p* < 0.0001 and β = −2.81512, *p* < 0.0001) for the AC and CC genotypes, respectively (Table 3). In women, coffee consumption and the rs1800588 genotypes were significantly associated with higher levels of HDL-C while the rs1800775 genotypes were significantly associated with lower levels of HDL-C (Table 3). The β-values were 0.81679; *p* = 0.0246 for coffee drinking, 0.99080; *p* = 0.0059 for the rs1800588 CT genotype, 3.16277; *p* < 0.0001 for the rs1800588 TT genotype, −1.80954; *p* < 0.0001 for the rs1800775 AC genotype and −2.81512; *p* < 0.0001 for the rs1800775 CC genotype (Table 3). Exercise, underweight and alcohol drinking were significantly (*p* < 0.001) associated with higher HDL-C levels (Table 2 and Table 3). However, vegetarian diet, overweight, obesity, smoking, second-hand smoke, WHR, TG and diabetes were significantly (*p* < 0.001) associated with lower levels of HDL-C (Table 2 and Table 3). After stratification by menopausal status, coffee drinking was significantly associated with higher HDL-C (β = 1.36554; *p* = 0057) only in non-menopausal women (Table 4). 

In the present study, the interaction between sex and coffee drinking on HDL-C was significant. After stratification by sex, coffee drinking was significantly associated with higher levels of HDL-C levels in women but not men. Moreover, after stratification by menopausal status, coffee drinking was significantly associated with higher levels of HDL-C levels in non-menopausal women but not menopausal women. To our knowledge, this is one of the first studies with a relatively larger sample size to assess the association between coffee drinking and HDL-C. Several potential confounders were adjusted including rs1800588 and rs1800775 variants, age, diet, exercise, BMI, smoking, tea and alcohol drinking, among others.

High-density lipoprotein cholesterol (HDL-C) is inversely associated with cardiovascular disease morbidity and mortality [1,4,5]. The positive association between coffee drinking and HDL-C that was observed in the present study implies that coffee might protect against atherosclerosis and other heart diseases. These results are in conformity with previous findings [27,28,32,33,34]. In addition, they somewhat conform to a study that demonstrated the anti-atherogenic property of coffee, where it enhanced HDL-mediated reverse cholesterol transport [7]. Reverse cholesterol transport is the main mechanism that helps in the removal of cholesterol from peripheral tissues and subsequent transport to the liver where it is reused or excreted as bile salts. By so doing, the cholesterol does not develop into foam cells, which are precursors of atherosclerotic plaques [6].

The antiatherogenecity of coffee is due to its possession of antioxidative phenolic compounds including quinides, chlorogenic and ferulic acids [7,35]. Moreover, coffee consumption has also been associated with markers of inflammation, which are well established predictors of heart disease risk [27,36,37].

The underlying mechanism for the presence of a significant association between coffee and HDL-C only in women especially non-menopausal women in the current study cannot be clearly stated. This could be due to hormonal differences between men and women as well as between menopausal and non-menopausal women [38]. For instance, the higher levels of HDL-C in non-menopausal women might be partly due to higher endogenous estrogen [12,39]. Coffee consumption and other caffeinated beverages have been reported to be beneficial to plasma levels of estrogen, sex hormone-binding globulin and estradiol in women [38,40]. Sex disparities in HDL-levels could also be due to physiological, physical and other lifestyle differences between men and women [38].

Our findings are not in line with those of some studies. For instance, an inverse association between coffee and HDL was reported [41]. In addition, even though coffee drinking was positively associated with HDL, an inverse association was observed in women after participants were stratified by sex [42]. In several other studies, coffee drinking was associated with lower HDL levels in women but not men [14,26]. Furthermore, several studies found no significant association between coffee drinking and HDL [29,30,43,44,45,46]. The inconsistent findings between the current study and those previously conducted could be due to different study populations, stratification of participants as drinkers or non-drinkers of coffee, sample sizes and the adjusted confounders. Comparatively, our study had a larger sample size and adjustments were simultaneously made for many potential confounders including rs1800588 and rs180077 genotypes, age, vegetarian diet, exercise, BMI, smoking, second-hand smoke, tea and alcohol drinking, WHR, LDL-C, TG and history of diabetes and hypertension.

The rs1800588 (LIPC) variant was associated with higher HDL-C levels in the present study. These findings are in line with others [17,19]. The LIPC gene possesses anti-atherogenic properties and the underlying mechanism is its ability to stimulate reverse cholesterol transport and remove intermediate-density lipoprotein (IDL) from circulation [21]. The rs100775 (CETP) variant was associated with lower levels of HDL-C levels in our study and other studies [17,18,20]. CETP is proatherogenic and promotes the transfer of cholesteryl ester from HDL-C to very low-density lipoprotein (VLDL) rich in triglycerides [22,23,24,47].

In a study conducted using the Taiwan biobank data, HDL was positively associated with age, exercise, underweight and current alcohol drinking and negatively associated with overweight, obesity, smoking, former alcohol drinking, WHR and vegetarian diet [34]. This is consistent with the findings of the present study. Similar associations of HDL with age, underweight, overweight, obesity, WHR, smoking, drinking and vegetarian diet have been previously reported [14,25,26].

The limitation of our study is that, the information on the type of coffee consumed by both men and women was not available in the Taiwan Biobank database. Therefore, we could not stratify the study participants based on the coffee type or drinking style.

## 4. Conclusions

Summarily, coffee drinking was significantly associated with higher HDL in women but not men after adjustments were made for confounders including rs1800588 (LIPC) and rs1800775 (CETP) single nucleotide polymorphisms. Therefore, coffee drinking might be cardioprotective especially in women.

## Figures and Tables

**Table 1:** Basic characteristics of male and female participants.

Variables	Men (*n* = 4262)	Women (*n* = 4813)	*p*-Value
HDL-C (mg/dL)	48.5852 ± 0.171	58.4673 ± 0.191	<0.0001
Coffee drinking			0.0018
No	2923 (68.58%)	3152 (65.49%)	
Yes	1339 (31.42%)	1661 (34.51%)	
rs1800588			0.1561
CC	1764 (41.39%)	1967 (40.87%)	
CT	1981 (46.48%)	2197 (45.65%)	
TT	517 (12.13%)	649 (13.48%)	
rs1800775			0.5161
AA	1034 (24.26%)	1217 (25.29%)	
AC	2194 (51.48%)	2435 (50.59%)	
CC	1034 (24.26%)	1161 (24.12%)	
Age (years)			0.6949
<49.10	2136 (50.12%)	2432 (50.53%)	
≥49.10	2126 (49.88%)	2381 (49.47%)	
Tea drinking			<0.0001
No	2352 (55.19%)	3362 (69.85%)	
Yes	1910 (44.81%)	1451 (30.15%)	
Vegetarian diet			<0.0001
No	4099 (96.18%)	4524 (94.00%)	
Yes	163 (3.82%)	289 (6.00%)	
Exercise			0.0314
No	2400 (56.31%)	2818 (58.55%)	
Yes	1862 (43.69%)	1995 (41.45%)	
BMI (kg/m^2^)			<0.0001
Underweight (BMI < 18.5)	1548 (36.32%)	2789 (57.95%)	
Normal (18.5 ≤ BMI < 24)	53 (1.24%)	181 (3.76%)	
Overweight (24 ≤ BMI < 27)	1589 (37.28%)	1140 (23.69%)	
Obesity (BMI ≥ 27)	1072 (25.15%)	703 (14.61%)	
Smoking			<0.0001
No	2422 (56.83%)	4597 (95.51%)	
Former	971 (22.78%)	101 (2.10%)	
Current	869 (20.39%)	115 (2.39%)	
Second hand smoke			<0.0001
No	3606 (84.61%)	4277 (88.86%)	
Yes	656 (15.39%)	536 (11.14%)	
Alcohol drinking			<0.0001
No	3413 (80.08%)	4713 (97.92%)	
Former	233 (5.47%)	33 (0.69%)	
Current	616 (14.45%)	67 (1.39%)	
Waist-hip ratio	0.8971 ± 0.001	0.8468 ± 0.001	<0.0001
LDL-C (mg/dL)	123.40 ± 0.488	120.30 ± 0.461	<0.0001
TG (mg/dL)	135.60 ± 1.537	101.80 ± 1.059	<0.0001
Diabetes			<0.0001
No	3718 (87.24%)	4423 (91.90%)	
Yes	544 (12.76%)	390 (8.10%)	
Hypertension			<0.0001
No	3211 (75.34%)	4110 (85.39%)	
Yes	1051 (24.66%)	703 (14.61%)	

Continuous variables are presented as means ± standard errors (SEs) while categorical variables are presented as percentages (%). *p* < 0.05—Significant; HDL-C—high-density lipoprotein cholesterol; LDL-C—low-density lipoprotein cholesterol and TG—triglyceride.

**Table 2:** Multiple linear regression showing the association between coffee drinking and high density lipoprotein cholesterol (HDL-C) in all participants.

Variables	β	*p*-Value
Sex (ref: Women)		
Men	−6.33560	<0.0001
Coffee drinking (ref: No)		
Yes	0.38920	0.1100
rs1800588 (ref: CC)		
CT	1.14306	<0.0001
TT	3.24816	<0.0001
rs1800775 (ref: AA)		
AC	−1.79140	<0.0001
CC	−2.71185	<0.0001
Age (ref: <49.10 years)		
≥49.10 years	1.36332	<0.0001
Tea drinking (ref: No)		
Yes	0.44026	0.0675
Vegetarian diet (ref: No)		
Yes	−5.33153	<0.0001
Exercise (ref: No)		
Yes	1.46267	<0.0001
BMI (ref: Normal)		
Underweight	5.47862	<0.0001
Overweight	−3.46621	<0.0001
Obesity	−5.09464	<0.0001
Smoking (ref: No)		
Former	−0.44759	0.2484
Current	−1.94760	<0.0001
Second hand smoke (ref: No)		
Yes	−0.36246	0.2863
Alcohol drinking (ref: No)		
Former	−0.29083	0.6731
Current	4.74316	<0.0001
Waist hip ratio	−23.34675	<0.0001
LDL-C	0.01325	0.0003
TG	−0.04312	<0.0001
Diabetes (ref: No)		
Yes	−2.02392	<0.0001
Hypertension (ref: No)		
Yes	−0.21625	0.4792

Interaction between sex and coffee *p*-value = 0.0452. *p* < 0.05—significant; HDL-C—high-density lipoprotein cholesterol; ref—reference; β—beta coefficient; LDL-C—low-density lipoprotein cholesterol and TG—triglyceride.

**Table 3:** Multiple linear regression showing the association between coffee drinking and HDL-C stratified by sex.

Variables	Men		Women	
	β	*p*-Value	β	*p*-Value
Coffee drinking (ref: No)				
Yes	−0.06663	0.8340	0.81679	0.0246
rs1800588 (ref: CC)				
CT	1.32430	<0.0001	0.99080	0.0059
TT	3.24976	<0.0001	3.16277	<0.0001
rs1800775 (ref: AA)				
AC	−1.96232	<0.0001	−1.80954	<0.0001
CC	−2.71536	<0.0001	−2.81512	<0.0001
Age (ref: <49.10 years)			0.0268	0.0371
≥49.10 years	0.54472	0.0991	2.47290	<0.0001
Tea drinking (ref: No)				
Yes	0.36651	0.2195	0.48684	0.1964
Vegetarian diet (ref: No)				
Yes	−4.75567	<0.0001	−5.50703	<0.0001
Exercise (ref: No)				
Yes	1.38414	<0.0001	1.63617	<0.0001
BMI (ref: Normal)			−4.6498	<0.0001
Underweight	7.72243	<0.0001	4.66897	<0.0001
Overweight	−3.68345	<0.0001	−3.06323	<0.0001
Obesity	−5.04663	<0.0001	−5.23881	<0.0001
Smoking (ref: No)				
Former	−0.21607	0.5638	−1.37464	0.2436
Current	−2.14450	<0.0001	−2.19635	0.0508
Second hand smoke (ref: No)				
Yes	−0.65050	0.1155	−0.06277	0.9076
Alcohol drinking (ref: No)				
Former	−0.45523	0.4916	0.73876	0.7186
Current	4.56251	<0.0001	5.19190	0.0003
Waist hip ratio	−23.64856	<0.0001	−23.21757	<0.0001
LDL-C	0.02023	<0.0001	0.00779	0.1591
TG	−0.03421	<0.0001	−0.05936	<0.0001
Diabetes (ref: No)				
Yes	−1.39585	0.0026	−2.21996	0.0007
Hypertension (ref: No)				
Yes	−0.43542	0.2256	0.34212	0.4998

Interaction between mean menopausal age and coffee *p*-value = 0. 0399. *p* < 0.05—significant; HDL-C—high-density lipoprotein cholesterol; ref—reference; β—beta coefficient; LDL-C—low-density lipoprotein cholesterol and TG—triglyceride.

**Table 4:** Linear regression showing the association between coffee drinking and HDL-C in women stratified by menopausal status.

Variables	<49.10 Years		≥49.10 Years	
	β	*p*-Value	β	*p*-Value
Coffee drinking (ref: No)				
Yes	1.36554	0.0057	0.06686	0.9013
rs1800588 (ref: CC)				
CT	1.25492	0.0127	0.78076	0.1307
TT	3.03425	<0.0001	3.37945	<0.0001
rs1800775 (ref: AA)				
AC	−2.65814	<0.0001	−0.99205	0.0902
CC	−3.54764	<0.0001	−2.08574	0.0023
Tea drinking (ref: No)				
Yes	0.65338	0.1930	0.23893	0.6762
Vegetarian diet (ref: No)				
Yes	−5.35759	<0.0001	−5.54455	<0.0001
Exercise (ref: No)			4.9049	<0.0001
Yes	1.27759	0.0174	1.91579	<0.0001
BMI (ref: Normal)			−4.8967	<0.0001
Underweight	3.02720	0.0052	8.31494	<0.0001
Overweight	−2.64081	<0.0001	−3.18604	<0.0001
Obesity	−5.71688	<0.0001	−4.58444	<0.0001
Smoking (ref: No)				
Former	−1.17161	0.4078	2.22341	0.2972
Current	−2.36302	0.0801	−1.41338	0.4893
Second hand smoke (ref: No)				
Yes	−0.11632	0.8641	0.07856	0.9301
Alcohol drinking (ref: No)				
Former	3.19330	0.3045	−0.94144	0.7330
Current	4.22680	0.0132	7.76976	0.0044
Waist hip ratio	−24.43827	<0.0001	−21.90699	<0.0001
LDL-C	0.00799	0.3393	0.01063	0.1594
TG	−0.06725	<0.0001	−0.05527	<0.0001
Diabetes (ref: No)				
Yes	−1.14892	0.4213	−2.50564	0.0008
Hypertension (ref: No)				
Yes	0.62290	0.5663	0.22589	0.6969

*p* < 0.05—significant; HDL-C—high-density lipoprotein cholesterol; ref—reference; β—beta coefficient; LDL-C—low-density lipoprotein cholesterol and TG—triglyceride.
